# RISK BEHAVIOR FOR BULIMIA AMONG ADOLESCENTS

**DOI:** 10.1590/1984-0462/;2019;37;2;00008

**Published:** 2019-02-25

**Authors:** Lorenna Mendes Temóteo Brandt, Liege Helena Freitas Fernandes, Amanda Silva Aragão, Thayná Pinto da Costa Luna, Rodrigo Macedo Feliciano, Sheyla Márcia Auad, Alessandro Leite Cavalcanti

**Affiliations:** a Universidade Estadual da Paraíba, Campina Grande, PB, Brazil.; b Universidade de São Paulo, São Paulo, SP, Brazil.; c Universidade Federal de Minas Gerais, Belo Horizonte, MG, Brazil.

**Keywords:** Adolescent behavior, Feeding and eating disorders, Feeding behavior, Adolescent health, Comportamento do adolescente, Transtornos da alimentação e da ingestão de alimentos, Comportamento alimentar, Saúde do adolescente

## Abstract

**Objective:**

To analyze the risk behavior for bulimia among female adolescents from public and private high schools.

**Methods:**

A cross-sectional study with a random sample of 850 female students aged 15-18 years was carried out in a city in northeastern Brazil, using the Bulimic Investigatory Test of Edinburgh (BITE) to assess the risk behavior for bulimia. Data were analyzed using the *Statistical Package for the Social Sciences* (SPSS) software and the Pearson’s chi-square , Fisher’s exact and robust Poisson regression tests, adopting the significance level of 5%.

**Results:**

Less than half of the sample (42.0%) showed standards of dietary risk and weight control practices; in 1.4% of the sample, bulimia signs were already installed. Fear of gaining weight was reported by 62.8% of the subjects. Risk practices were lower among students from public schools; (*Odds Ratio* - OR - 0.82; confidence interval of 95% - 95%CI - 0.69-0.97). Among restrictive practices, fasting for a whole day was the most applied (29.9% of the students). Among individuals who were at risk situation, almost half believed to have normal eating habits (prevalence *ratio* - PR - 0.42; 95%CI 0.36-0.49). Individuals who consider their eating habits normal, who are afraid of gaining weight, those who seek emotional comfort in food and follow strict diets had higher risk for bulimia (p<0.05).

**Conclusions:**

The number of female adolescent students with risk behavior practices for bulimia is high, and the frequency of those unaware of this situation is also very high. Risk situations emerge as a collective health problem, and individuals from private schools were more likely to be in this situation.

## RESUMO

**Objetivo:**

Analisar comportamentos de risco para bulimia em adolescentes do sexo feminino de escolas públicas e particulares.

**Métodos:**

Estudo transversal com amostra aleatória de 850 estudantes do sexo feminino, com idades entre 15 e 18 anos, realizado em cidade do Nordeste do Brasil, utilizando o *Bulimic Investigatory Test of Edinburgh* (BITE) para avaliar comportamentos de risco para transtornos alimentares. Os dados foram analisados utilizando o teste do qui-quadrado de Pearson, o teste exato de Fisher e a regressão de Poisson, com o *software Statistical Package for the Social Sciences* (SPSS), adotando o nível de significância de p<0,05.

**Resultados:**

Da amostra estudada, 42,0% apresentou padrões de risco e práticas de dieta e controle de peso e 1,4% já apresentava sinais de bulimia instalados. O medo de ganhar peso foi relatado por 62,8% das adolescentes. As práticas de risco foram menos frequentes em estudantes de escolas públicas (*Odds Ratio* - OR - 0,82; intervalo de confiança de 95% - IC95% - 0,69-0,97). Entre as práticas restritivas, jejum por um dia inteiro foi o mais aplicado pelas participantes (29,9%). Entre os indivíduos com situação de risco, quase metade acreditava ter hábitos alimentares normais (razão de prevalência - RP - 0,42; IC95% 0,36-0,49). Estudantes que consideram seus hábitos alimentares normais, que têm medo de ganhar peso, que procuram conforto emocional em alimentos e seguem dietas rigorosas tiveram maior risco para bulimia (p<0,05).

**Conclusões:**

O número de estudantes com práticas de comportamento de risco para bulimia é alto, e o número daquelas que desconhecem essa situação também é muito alto. As situações de risco emergem como problemas de saúde coletiva, e indivíduos de escolas particulares são mais propensos a apresentar transtornos alimentares.

## INTRODUCTION

Eating disorders are behavioral syndromes that have gradually aroused interest in the scientific community over the last 30 years, as they can lead to poor quality of life (QOL),^1^ they are associated with different types of eating-related behavior and may affect individuals in various ways, severity and intensity.^2^ These disorders, especially anorexia nervosa and bulimia nervosa, have been reported in young women,^3^ with onset peak between 14 and 19 years,^4^ because this is a period prone to dissatisfaction with body shape and construction of identity.^5^ Although surveys have shown the presence of these disorders in men^6^ - previously underestimated -, these diseases have a clear predominance among females.^5^

Several reasons have been attributed to the development of eating disorders, and most of them are psychosocial.^3^ The overvaluation of thinness, which is commonly seen in women, is considered as a major contributing factor in the pathogenesis of eating disorders,^3^ allied to the media that promote the culture of beauty, maintained by a “beauty industry”,^7^ ^,^ ^8^ which associates thinness to an image of success. This context leads to the so-called “inappropriate behaviors and practices for weight control,” especially among adolescents.^7^ ^,^ ^8^ Genetic predisposition^5^ is also pointed and, in combination with other specific physical or psychological stressors, may place an individual at higher risk of developing an eating disorder.^9^

The genesis and maintenance of eating disorders are multifactorial, involving biological, social and psychological factors, with a key point at the distorted self-perception and dissatisfaction with physical appearance.^1^ ^,^ ^10^ The number of adolescents with eating disorders is growing, driven by the fear of getting fat, use of prohibitive diets or inappropriate methods of food excess compensation, such as diuretics and/or laxatives and self-induced vomiting.^11^ These facts have resulted in increased prevalence of eating disorders over the recent years:^12^ anorexia nervosa ranges from 0.3 to 3.7%; and bulimia nervosa, from 1.1 to 4.0% in girls.^13^

Therefore, eating disorders are classified as the third most common chronic disease in adolescents, especially among women,^14^ ^,^ ^15^ and are associated with morbidity and mortality rates that are among the highest of all mental disorders,^14^ ^,^ ^16^ with significant functional impairment,^16^ with lower weighted mortality ratios in bulimia nervosa: 1.93 per 1,000 person-year;^17^ and higher in anorexia nervosa: 5.86 deaths per 1,000 person-year.^17^ However, despite the high severity and high chronicity of the disease,^4^ this topic remains relatively not as studied as the other mental disorders.

The development of studies of risk groups improves the analysis of their vulnerability and helps the design and establishment of preventive measures to avoid consequent comorbidities.^2^ The need to perform more studies on eating disorders associated with risk factors has been emphasized in literature,^8^ ^,^ ^15^ ^,^ ^18^ ^,^ ^19^ because the knowledge generated by these studies could guide the development of education programs addressing food intake, obesity and prevention of eating disorders.^18^

Due to the importance of this disease, as well as the lack of recent Brazilian studies addressing this issue - especially in the northeastern region, besides the disagreement of the results observed in the few studies already done -, we aimed to investigate the relationship between risk behaviors for bulimia in female adolescents from public and private high schools with inadequate weight control practices and sociodemographic factors.

## METHOD

This cross-sectional study was carried out with female students from 14 public and private high schools previously drawn in Campina Grande, Paraíba, Brazil, a city in northeastern Brazil with about 402,912 inhabitants and human development index (HDI) of 0.72.

The population of this study totaled 14.351 adolescents, and the sample size was calculated using the error margin of 1%, confidence level of 95% and prevalence of risk behaviors for eating disorders of 1.7%.^15^ The correction factor of 1.2 was applied, due to the multistage feature of the study. The minimum sample size needed to meet the requirements was estimated at 780 individuals. However, an additional 10% was invited to participate in the study, in order to offset potential refusals, totaling 858 individuals. At the end, the sample of this study consisted of 850 individuals. The proportion of students from public and private schools that were part of the sample was considered, respecting the percentage of students aged 15-18 enrolled in the public and private network of the municipality distributed in health districts. Adolescents already diagnosed with eating disorders or gastroesophageal reflux were excluded from this study.

This research followed the ethical principles established by Brazilian and international legislation and was approved by the Ethics Committee on Human Research of the State University of Paraíba (Universidade Estadual da Paraíba - UEPB). All students were invited to participate through signing the informed consent form by parents or guardians and signing themselves the consent term, as established by Resolution nº 466/2012 of the National Health Council.

A pilot study was conducted in November 2014 with 59 students from a public school to test the methods and the data collection process, demonstrating that there was no need for modification. Individuals who participated in this stage were not included in the main study.

Initially, a visit was held in each classroom informing about the objectives and the importance of the research. At this time, the informed consent form and the consent term were delivered to the adolescents interested in participating in the survey. During the next day, a questionnaire with socioeconomic and demographic variables (age and school type) and the Bulimic Investigatory Test of Edinburgh (BITE)^20^ were applied. BITE was validated to be applied in Brazilian adolescents.^21^

The BITE presents as final results two scales, one of symptoms and other of severity. The scale of symptoms has three possible outcomes:

- Situation of “no risk”, for the development of eating disorders (bulimia) (score <10).
- “Risk situation” (score ≥10 and <20), for the development of eating disorders, which suggests an unusual eating standard without any of the criteria that characterize an eating disorder.
- “Eating disorder situation” (score from 20 to the maximum of 30), characterized by the presence of binge-eating behavior and high possibility of bulimia, which is considered the main indicator for the occurrence of eating disorders.

The gravity scale has, as well, three possible outcomes:

- Mild gravity (<5 points).
- Moderate gravity (5-9 points).
- High gravity (≥10 points).

It is recommended that subjects respond to the questionnaire considering their behavior during the last three months.^20^

Descriptive statistics were used to characterize the sample. Bivariate analyses were performed to test the association between risk behaviors for bulimia and behavioral and sociodemographic variables, and to evaluate the possible association between school type and behavioral methods to lose weight, using the exact versions of the Pearson’s chi-square and Fisher’s exact tests. The Poisson regression with robust variance was used to verify the association of behavioral and sociodemographic variables with behavioral risk for bulimia. The level of statistical significance was set at 5%, with 95% confidence interval (95%CI).

## RESULTS

The sample consisted of 850 students (34.1% was 15 years-old, 33.1% 16 years-old, 23.2% 17 years-old, and 9.6% 18 years-old), and most of them (76.2%) were students from public schools.

Regarding BITE results, the symptom scale identified 42.0% of adolescents in behavioral risk for bulimia, *i.e.*, they had an unusual dietary standard and used harmful practices for weight control. On the same scale, 4.8% of adolescents achieved ≥20, indicating high probability of presenting bulimia nervosa. Of these, 1.4% reached the highest cut-off points in both symptom and severity scales, indicating not only the high possibility to meet the bulimia nervosa criteria, but also the presence of high-severity risk behavior.

Among the restrictive practices, fasting for a whole day was the most used method among adolescents (29.9%), and the distribution of this practice among public and private schools was similar (p-value=0.26) (Table 1). In the bivariate analysis between school type and behavioral variables, only the use of laxatives was associated with school type (p-value<0.05). However, all variables with p-value<0.20 were added to the multivariate model, as well the “use of pills” and “fasting” for their epidemiological importance. In the final robust Poisson regression model (Table 1), school type was associated with the use of laxatives (prevalence *ratio* - PR - 0.89; 95%CI 0.81-0.98) and self-induced vomiting (PR 1.0; 95%CI 1.0-1.1).

**Table 1:** Distribution of behavioral methods to lose weight in the bivariate analysis and multivariate model (Poisson regression), according to school type.

	Independent variable - n (%)		Dependent variable		
	School		p-value (bivariate)	p-value (Poisson’s regression)	Adjusted PR (95%CI)
	Public	Private			
Use of pills					
No	602 (76.4)	186 (23.6)	0.69	0.78	-
Yes	46 (74.2)	16 (25.8)			
Use of diuretics					
No	627 (76.7)	191 (23.3)	0.15	0.40	-
Yes	21 (65.6)	11 (34.4)			
Use of laxatives					
No	609 (77.2)	180 (22.8)	0.01	0.02	0.89 (0.81-0.98)
Yes	39 (63.9)	22 (36.1)			
Self-induced vomiting					
No	546 (75.1)	181 (24.9)	0.05	0.01	1.08 (1.01-1.15)
Yes	102 (82.9)	21 (17.1)			
Fasting					
No	448 (75.2)	148 (24.8)	0.26	0.50	-
Yes	200 (78.7)	54 (21.3)			

PR: prevalence *ratio*; 95%CI: confidence interval of 95%.

In relation to the occurrence of binge-eating behavior indicators, 34.5% of the studied adolescents eat until feeling physically ill, and 36.6% of them feel guilty for eating too much. Almost half of the adolescents feel an uncontrollable urge to eat (48.5%) or eat large amounts of food in a short period of time (45.2%). Only a small portion (10.4%) considered themselves compulsive eaters (Table 2).

**Table 2:** Frequency of episodes of binge-eating among adolescents.

Compulsive eating indicators	n	%
Eats until feeling physically ill		
No	557	65.5
Yes	293	34.5
Total	850	100.0
Feels an uncontrollable urge to eat		
No	438	51.5
Yes	412	48.5
Total	850	100.0
Eats large amounts of food in a short period of time		
No	466	54.8
Yes	384	45.2
Total	850	100.0
Feels guilty for eating too much		
No	538	63.4
Yes	311	36.6
Total	849	100.0
Considers herself a compulsive eater		
No	762	89.6
Yes	88	10.4
Total	850	100.0

In the multivariate model (Table 3), that analyzes behavioral and socioeconomic factors, all variables remained associated with behavioral risk for bulimia in the final model: adolescents who consider having normal eating habits (PR 0.42; 95%CI 0.36-0.49); fear of getting fat (PR 0.61; 95%CI 0.51-0.75); searching emotional comfort in food (PR 0.55; 95%CI 0.48-0.64), fasting for a whole day (PR 0.76; 95%CI 0.66-0.88); following a strict diet (PR 0.70; 95%CI 0.58-0.85); as well as the variable school type. The proportion of adolescents who presented risk behaviors was significantly lower in public schools (PR 0.82; 95%CI 0.69-0.97) compared to adolescents from private schools.

**Table 3:** Distribution of behavioral risk for eating disorders in the bivariate and multivariate analysis, according to behavioral aspects and school type.

	Independent variable - n (%)		Dependent variable		
	Risk		p-value (bivariate analysis)	p-value (Poisson’s regression)	Adjusted PR (95%CI)
	Absent	Present			
Level 1) Behavioral characteristics					
Believes to have normal eating habits					
No	42 (18.7)	183 (81.3)	0.001	0.001	0.42 (0.36-0.49)
Yes	451 (72.2)	174 (27.8)			
Fear of getting fat					
No	238 (75.3)	78 (24.7)	0.001	0.001	0.61 (0.51-0.75)
Yes	255 (47.8)	279 (52.2)			
Searches for emotional comfort in food					
No	415 (68.5)	191 (31.5)	0.001	0.001	0.55 (0.48-0.64)
Yes	78 (32.0)	166 (68.0)			
Fasting for a whole day					
No	392 (65.8)	204 (34.2)	0.001	0.001	0.76 (0.66-0.88)
Yes	101(39.8)	153 (60.2)			
Follows strict diet					
No	475 (60.5)	310 (39.5)	0.001	0.001	0.70 (0.58-0.85)
Yes	18 (27.7)	47 (72.3)			
Level 2) Socioeconomic features					
School type					
Public	389 (60.0)	259 (40.0)	0.03	0.02	0.82 (0.69-0.97)
Private	104 (51.4)	98 (48.6)			

PR: prevalence *ratio*; 95%CI: confidence interval of 95%.

## DISCUSSION

Evidence points to an increasingly wide distribution of eating disorders worldwide.^22^ Being female and adolescent do trigger eating disturbances and risk of eating disorders.^23^ Our results show that almost half of the studied adolescents was at risk for bulimia, which is considered high. Other studies using the same data collection instrument, but addressing different age groups in adolescence, also found high prevalence of risk behavior among Brazilian adolescents: 40.0,^8^ 25.2,^11^ 36.5,^24^ and 41.7%.^15^ Among high school female students, eating disorders are estimated to occur anywhere at rates between 15.8 and 57%.^25^ ^,^ ^26^ It was also observed that 1.4% of the studied female adolescents have strong indications that they are already suffering from bulimia. The results found in this study are in line with the literature for studies with Brazilian subjects: 1.1,^6^ 1.2,^8^ and 1.7%.^15^

In this study, the proportion of adolescents who presented risk practices was significantly higher in private schools compared to adolescents from public schools, and this variable remained associated with behavioral variables in the final multivariate analysis model. This same finding was observed in another Brazilian study:^8^ attending a private school appeared to be a condition that increases the risk of abnormal eating behaviors (p-value<0.001). However, this finding differs from Jordanian researchers,^23^ who observed no association between the type of school and risk behaviors for eating disorders (p-value>0.05). A Brazilian study developed by Hermont et al.^15^ did not verify this association neither, but it found proportionally more cases of risk behavior among students from private schools compared to those from public schools. Thus, it is clear that the desire to be thin is similar in both type of schools, but adolescents from private schools use improper practices to achieve this desire at a higher frequency compared to those from public schools, probably due to the greater access to these resources, which are often expensive,^8^ and probably due to greater disclosure of inappropriate methods for weight control among adolescents of private schools. According to American researchers,^27^ peer pressure to conform to cultural ideals has been consistently identified as an important factor associated with the development of eating disorders, especially among adolescents. Adolescent girls are subject to a number of physical, psychological, and social changes that, if not managed well, have negative impacts on their self-esteem, body shape satisfaction, and psychological well-being.^23^

Among the restrictive practices, fasting for a whole day was the most applied among adolescents (29.9%), and the distribution of this practice among public and private schools was similar, corroborating the results obtained by Vale, Kerr and Bosi.^8^ Evaluation of the spread of restrictive practices by adolescents according to school type showed a significant higher use of laxatives among adolescents from private schools. In the multivariate analysis, both the use of laxatives and the practice of self-induced vomiting remained in the final model, and the practice of self-induced vomiting showed strong association with adolescents from public schools. These results indicate that the access to financial resources can influence the behavioral method used to lose weight by adolescents. Vale, Kerr and Bosi^8^ suggested that most adolescents who perform self-induced vomiting, fast or eat compulsively believe to have normal eating habits, suggesting an association between leanness and health, perhaps as a contrast to the association between obesity and disease. In this study, almost half of adolescents with behavioral risk for bulimia believed to have normal eating habits, which is concerning, as it demonstrates a trend towards lack of awareness and/or self-denial of their own condition. Japanese researchers^28^ point out that the participants of these studies may be reluctant to admit such behaviors; and this may be linked to self-denial of the condition, guilt, or shame of their behavior, in order to hide the true source of the problem.^16^

According to Vale, Kerr and Bosi,^8^ eating and restricting food appeared to be harmful strategies to deal with conflicting situations and their aversive resulting emotional states. In this study, the prevalence of adolescents who reported fear of getting fat was high, and more than half of these individuals presented behavioral risk for bulimia. Similarly, over half of individuals who claimed to seek for emotional comfort in food were at risk for eating disorders. The conflict between food and anxiety can lead to the emergence of food compulsion and obesity. The psychosomatic theory of obesity states that, in times of distress, food is used as an emotional defense that, in turn, leads to obesity,^29^ and that obese people consume large amounts of food in response to negative emotions, while people with normal weight have a more adaptive coping mechanisms and do not eat in response to emotional stress.^30^ However, in a study conducted in the United States,^31^ not all negative effects lead to excess eating in response to emotional conditions in adolescents. This may possibly provide a focus of intervention in this population. These findings support potential implications for the treatment and prevention of pediatric obesity and eating disorders, because they suggest that interventions would benefit the incorporation of stress-reduction techniques and promotion of positive mood.

Behaviors indicative of binge eating showed high prevalence in this study, well above the purgative restrictive practices and all variables remained in the final multivariate analysis model associated with behavioral risk for bulimia. According to the fifth edition of Diagnostic and Statistical Manual of Mental Disorders (DSM-5), a binge-eating episode is characterized by both the intake of an amount of food that would normally be considered too large and the feeling of lack of control over eating.^10^ According to Italians researchers,^32^ what initially occurs is the lack of self-control, leading the patients to abandon their efforts to restrict food, resulting in a bulimic episode. This intensifies their concerns about their inability to control feeding and weight, and encourages the adoption of dietary restriction practices, which, in turn, increases the risk of binge eating, creating a cycle.

The prevalence of adolescents who follow a strict diet was low in our sample (7.6%). However, of them, 72.3% had behavioral risk for bulimia. Diets cause a weight fluctuation experience and may provoke depression and risk of developing eating disorders.^33^ Therefore, feeding is an issue that requires special attention, especially in the field of public health,^8^ because following a diet does not guarantee weight loss and cannot be part of a healthy lifestyle.^33^ The high number of adolescents with inadequate dietary practices in this study highlights the attention that this issue requires from the academic community, especially in the field of public health, particularly in Brazilian health scenario.^8^ Overall, these results will help health professionals and researchers to interpret the risk as a strong ally in the prevention of diseases such as bulimia, which can cause severe psychological and physical damage to individuals and may also result in death.

The findings in this report are subject to some limitations. Firstly, the cross-sectional design impeding any evaluation of causality. Further longitudinal studies are required to overcome this limitation. Secondly, the standardization of the indices would facilitate the comparisons of different studies. Despite these limitations, the development of studies that assess risk groups, especially in adolescence - a trigger point in the development of diseases such as eating disorders -, is important for the evaluation of their vulnerability, as well as for establishing preventive measures to avoid the perpetuation of inappropriate habits, thus avoiding comorbidities, which can last for a lifetime.

The number of individuals with behavioral risk practices for bulimia and the number of individuals who are unaware of their problem are very high. Studies that work with risk data point out the longitudinal follow-up of participants with high scores on self-reporting instruments, aiming to implement measures to minimize the damage, reverse the situation and mitigate the potential impact of eating disorders on the physical and psychological aspects and welfare. Risk situations emerge as a collective health problem, and, in this study, students from private schools were more likely to be found in this situation.
